# Kinetic Modeling and Assessment of a CO_2_ Nanobubble-Enhanced Hydrate-Based Sustainable Water Recovery from Industrial Effluents

**DOI:** 10.1007/s40831-025-01081-8

**Published:** 2025-04-22

**Authors:** Seyed Mohammad Montazeri, Nicolas Kalogerakis, Georgios Kolliopoulos

**Affiliations:** 1 Department of Mining, Metallurgical, and Materials Engineering, Université Laval, Québec, QC G1V 0A6 Canada; 2 School of Chemical and Environmental Engineering, Technical University of Crete, 73100 Chania, Greece

**Keywords:** CO_2_ hydrate-based desalination, Effluent treatment, Water recovery, Nanobubbles, Kinetic modeling, Kinetic promoter, Mineral processing, Hydrometallurgy

## Abstract

This study evaluates the effectiveness of CO_2_ nanobubble-enhanced hydrate-based desalination (HBD) to treat industrial effluents from the mining and metals industry. Testing was conducted in a high-pressure reactor apparatus that employed CO_2_ as the gas hydrate former at 274.15 K and 3.58 MPa. CO_2_ nanobubbles (NBs) were used to promote hydrate formation, aiming to streamline an HBD process without separation steps for the additives/chemicals used. Due to the limited studies on hydrate formation in sulfate-containing aqueous solutions, this research focused on the kinetics of hydrate formation in varying concentrations of Na_2_SO_4_ and MgSO_4_ (0.1 and 0.5 M). The results showed that CO_2_ NBs significantly enhanced hydrate formation in both Na_2_SO_4_ and MgSO_4_ solutions, with CO_2_ consumption increasing by up to approximately 51% and 35%, respectively. Additionally, a kinetics study on a real effluent from the mining and metals industry showed that the presence of CO_2_ NBs increased CO_2_ consumption by around 20% after 180 min. This research also evaluated water recovery and desalination efficiency in a 3-stage HBD process applied to the effluent, the concentration of which exceeded the operating range of reverse osmosis. The results indicated an improvement in water recovery from 25.13 ± 2.04% to 40.16 ± 1.43% with CO_2_ NBs, underscoring their effectiveness in treating highly saline water. Moreover, desalination efficiencies of 49.54 ± 2.39% and 42.03 ± 3.43% were achieved without and with CO_2_ NBs, respectively. This study represents the successful demonstration of the efficient application of the CO_2_ NBs-boosted HBD method to treat high-salinity effluents and recover clean water for reuse.

**Graphical Abstract:**

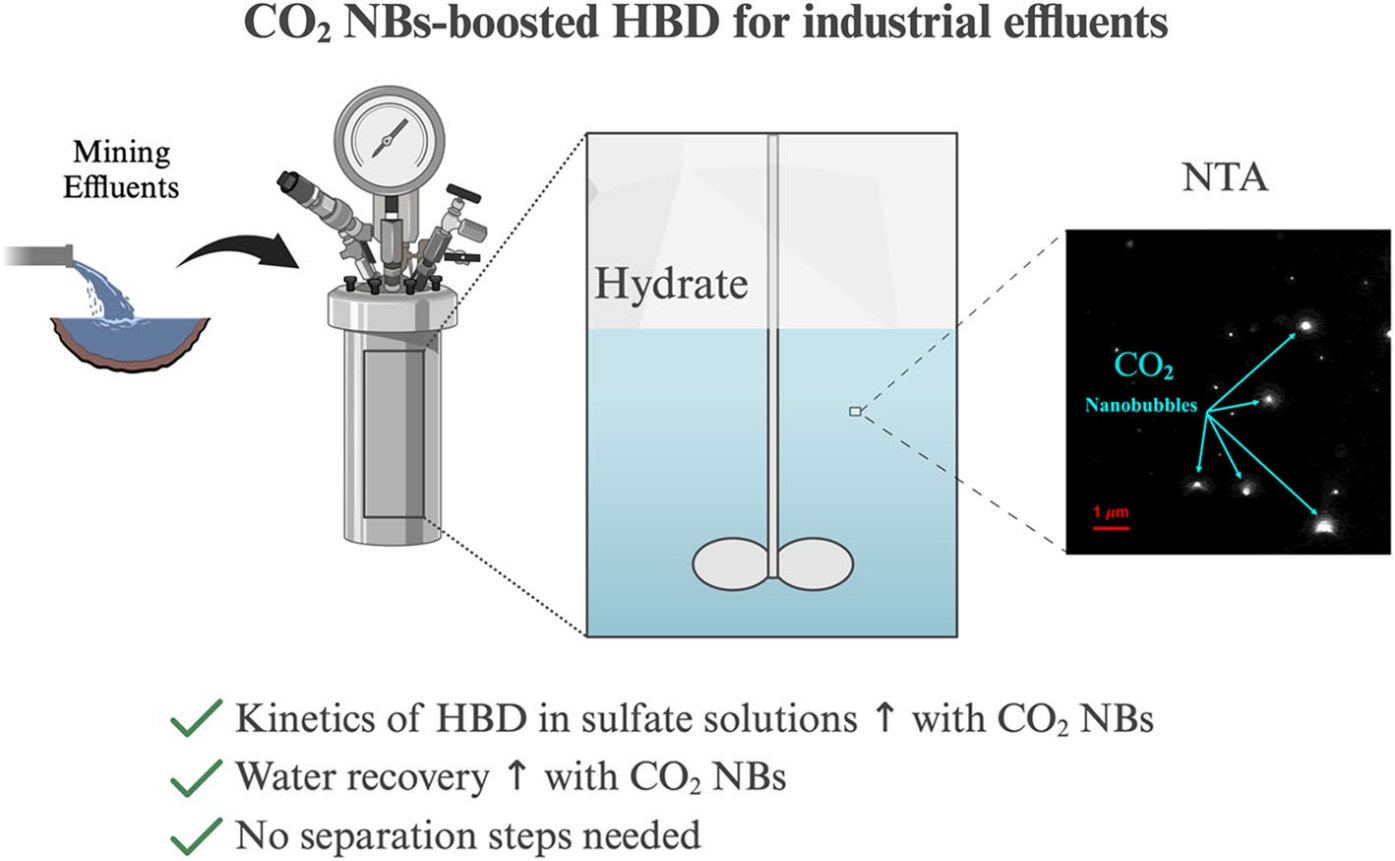

## Introduction

The shortage of freshwater resources underscores the importance of water purification to ensure a supply of high-quality drinking water for people and usable water for industry [1]. The primary sources of contamination in water reservoirs come from industrial and municipal facilities releasing wastewater that has not been adequately treated. For instance, mining, mineral, and metal processing results in the discharge of substantial volumes of industrial wastewater (i.e., effluents) [2]. Water from mines typically contains high levels of sulfates and different ions. These water sources are also known for having high mineralization and increased hardness [3]. Sulfate concentrations surpassing 1000 ppm result in unfavorable results, such as triggering the release of phosphates from sediment layers [3]. Nowadays, the natural resources industry faces challenges such as limited water and energy resources, depletion of high-grade mineral deposits, and environmental concerns, all while having to drastically increase mining of certain elements to meet the skyrocketing demand during the energy transition to the electric era [4]. Accordingly, the limited availability of freshwater and the growing demand for it highlight the urgency to create cost-effective and efficient methods for clean water recovery and distribution, such as desalination technologies [3, 5, 6].

Desalination can separate salts from saline water to generate clean water [5, 7]. It is currently reported that water desalination worldwide has reached a volume of 95 million m^3^ per day [1, 8]. In recent years, there has been a significant shift in the water market from thermal desalination to membrane-based desalination, particularly reverse osmosis (RO) [9]. The move away from thermal methods is primarily due to their substantial energy requirements, demanding both thermal and mechanical energy sources [6, 9–11]. However, RO is vulnerable to industrial multi-component effluents found in mineral and metals industry [12, 13], oil and gas facilities, and textile and food industries [9], as RO treatment of these solution leads to membrane fouling problems [5]. Also, various chemicals are commonly used during the pre-treatment stage of RO to reduce corrosion, adjust pH, and chlorinate the water, generating RO brines with residual chemicals that often raise concerns [1, 14].

Hydrate-based desalination (HBD) is as an energy-efficient approach to recover clean water from low- to high-salinity aqueous solutions, thus decreasing brine discharge and limiting environmental concerns [5, 15]. HBD uses the formation of gas hydrates, where water crystallizes at temperatures above its normal freezing point, and is considered a relatively green process [6, 16]. These gas hydrates are formed when water molecules create structured cages around guest gas molecules, like CO_2_ or CH_4_, through hydrogen bonding under specific temperature and pressure conditions [17–22]. Gas hydrate formation is a selective process, which excludes any dissolved ions from the resulting crystals [23, 24]. By separating the gas hydrates from the brine, i.e., through filtration, and decomposing the gas hydrate crystals, i.e., upon exposure to atmospheric conditions, heat stimulation, or a decrease in pressure, salt-free water and the guest gas molecules can be reclaimed for reuse in various industrial sectors, including the mining and metals industry [6, 22]. HBD is an energy-efficient process with operating pressures for seawater desalination being generally about half that of the RO process [6, 25–29]. Thus, HBD offers improved efficiency for sustainable and scalable implementations, addressing the main limitations of traditional desalination techniques [30].

HBD, despite its potential advantages, faces challenges particularly regarding slow hydrate formation kinetics, which can impede its widespread industrial adoption [17, 31]. Researchers have been actively investigating non-toxic, cost-effective, and eco-friendly additives to facilitate the formation of gas hydrates in HBD [6]. However, traditional gas hydrate formation promoters, like sodium dodecyl sulfate (SDS) and tetrahydrofuran (THF), are unsuitable for HBD applications due to issues like foam formation and toxicity, while nanoparticles introduce extra processing steps,

i.e., separation, thus driving up the overall desalination cost [6, 32–34]. Moreover, the absence of experimental studies on effluent desalination using HBD, particularly those containing sulfates, such as mining, mineral, and metal processing effluents, is a significant limitation. Addressing this gap in research is crucial for further advancement and industrial implementation of HBD.

This study investigated the kinetics of HBD using aqueous solutions containing high and low concentrations of N_2_SO_4_ and MgSO_4_ (0.1 and 0.5 M), typical components found in effluents from the mining and metals industry, for the first time. CO₂ was selected as the gas hydrate former due to its non-toxic and non-flammable properties as well as its potential role in reducing the carbon footprint through capture and reuse in industrial applications [30, 35]. Additionally, this work examined the influence of CO_2_ nanobubbles (NBs) on the kinetics of HBD in sulfate solutions, serving as an innovative kinetic promoter, and developed a kinetic model of CO_2_ hydrate formation in the presence of nanobubbles. NBs, with diameters less than 1 μm, demonstrate unique physicochemical characteristics such as reactivity and longevity in aqueous environments [36–39]. They provide benefits such as facilitating CO_2_ hydrate formation and eliminating the need for separation of the kinetic promoter from the recovered water. Furthermore, the kinetics of HBD using a real effluent received from a mining and metals company in Québec, Canada, were examined with and without the presence of CO_2_ NBs. Subsequently, the potential of HBD to recover water from this effluent was experimentally assessed in a three-stage process. Key desalination parameters such as hydrate conversion, water recovery, and desalination efficiency were measured. The findings indicate that the inclusion of NBs notably improves both the kinetics of hydrate growth and the amount of water recovered, which reached 40.16 ± 1.43%, while causing only a minimal decrease in the desalination efficiency. Our findings, particularly for the use of CO_2_ NBs in HBD, provide useful insights into the development of sustainable zero liquid discharge technologies for the mining, mineral, and metal processing industry worldwide.

## Methodology

### Materials

High-purity carbon dioxide gas (CO_2_, 99.995%, Praxair Canada Inc.) served as both the gas hydrate former and the source for generating CO_2_ NBs. Sodium sulfate (Na_2_SO_4_ anhydrous, > 99%) and magnesium sulfate (MgSO_4_ anhydrous, > 99%), purchased from Fisher Scientific, were utilized in preparing the saline aqueous solutions. These solutions were prepared using ultrapure water (18.2 MΩ cm) obtained from a Millipore Milli-Q water purification system. The effluent sample, whose composition is detailed in Table 1, was received from a mining and metals company in Québec, Canada.

**Table 1:** Concentration of major components in the effluent tested in this work

Elements	Na^+^	K^+^	Mg^2+^	Ca^2+^	Cl^−^	SO_4_^2−^
Concentration (ppm)	15,800	504	2110	2070	34,000	3500

pH 6.71/Electrical conductivity = 91.05 mS/cm

### Generation and Analysis of CO_2_ NBs

Using an MK1 NanoBubbler™ from Fine Bubble Technologies Ltd., Cape Town, South Africa, which is constructed from stainless steel grade 316, CO_2_ NBs were produced in ultrapure water. The MK1 operated continuously for 75 min, maintaining a CO₂ flow rate of 30 standard liters per minute, with the gas cylinder outlet pressure set to 0.07 MPa using a gas regulator. The nanobubbler functions by sucking in CO₂ gas, which passes through a venturi nozzle, generating microbubbles in water. This microbubble–water mixture is then directed into a cavitation cylinder, where shear forces and pressure variations further break down the microbubbles into nanobubbles. To assess the size and concentration of the CO_2_ NBs formed, Nanoparticle Tracking Analysis (NTA) was conducted using a ZetaView® BASIC NTA device from Particle Metrix, Germany. NTA utilizes visualization techniques considering light scattering and the Brownian motion of dispersed particles to derive particle size distributions in solution [37, 40]. Calibration of the NTA instrument was performed using a 250 000-fold dilution of Nanosphere Standards (Thermo Scientific, #3100A) in ultrapure water, with all measurements being conducted at least in triplicate for reliability and accuracy.

### Desalination Equipment

**Fig. 1:**
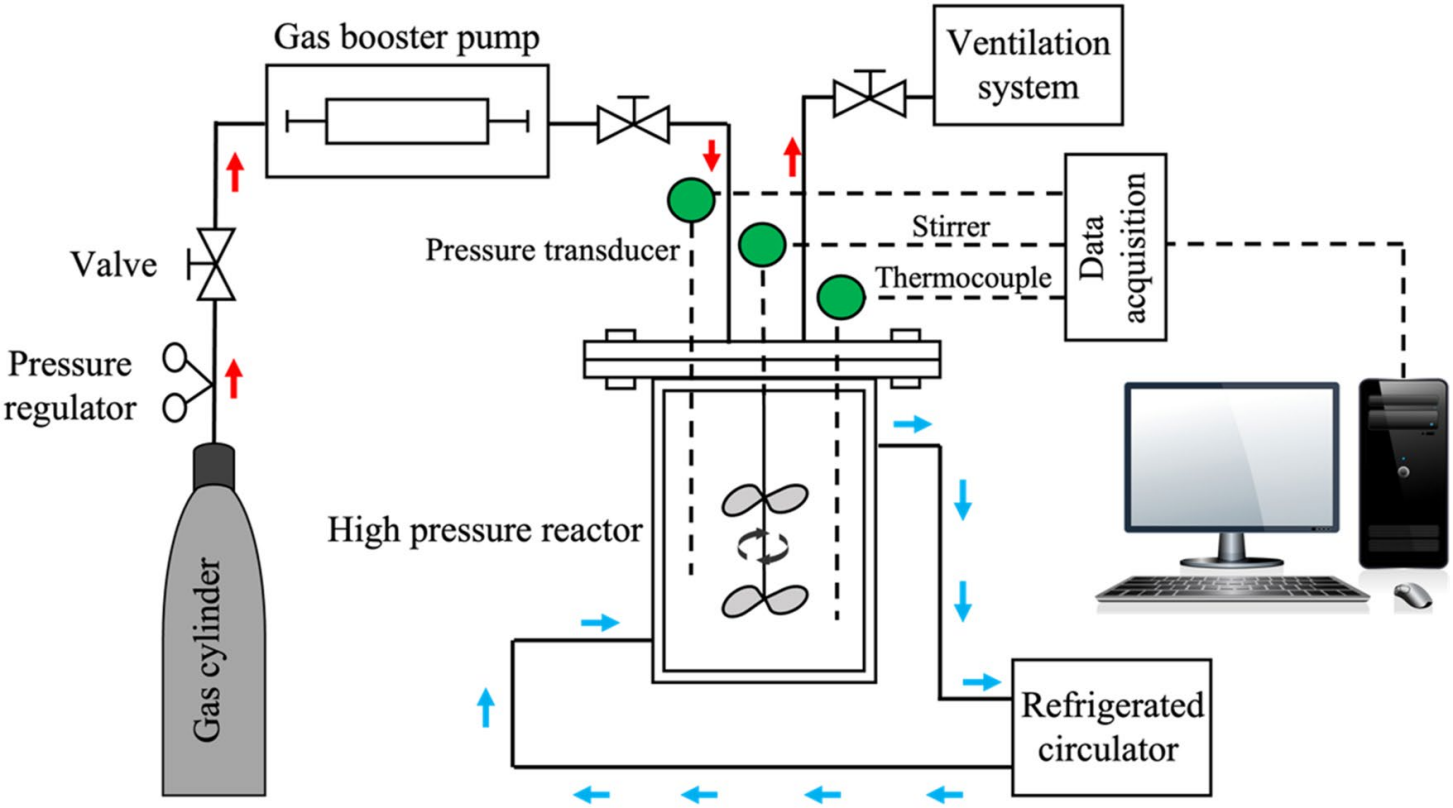
Schematic of the apparatus used for the CO_2_ gas hydrate-based desalination experiments including high pressure reactor, gas booster pump, refrigerated circulator, and data acquisition system (reproduced from [5])

Figure 1 illustrates the experimental setup used for the HBD experiments, showing a 1.8 L jacketed high-pressure stirred reactor made of Hastelloy C-276, provided by Parr Instrument Company. CO_2_ gas was supplied from a pressurized cylinder and passed through a gas booster pump from Haskel International, Inc., which can achieve an outlet pressure of up to 62 MPa, before entering the reactor. Temperature control was managed using a refrigerated circulator from VWR International, LLC, with a refrigerant mixture of water and ethylene glycol (60–40 vol.%). To ensure thorough mixing of CO_2_ gas and water, an overhead stirrer operated at 300 rpm. The setup employed valves to maintain constant pressure; these valves were opened manually whenever the pressure dropped below the desired level due to CO_2_ consumption. Continuous monitoring of the reactor's conditions was performed using temperature detectors and pressure transducers. Data collection was facilitated by a 4848 reactor controller data acquisition unit from Parr Instrument Company. A detailed description of the HBD apparatus is available in Montazeri et al. [5].

### Desalination Methodology

Saline aqueous solutions of Na_2_SO_4_ and MgSO_4_ with concentrations of 0.5 M and 0.1 M were prepared by dissolving the appropriate amounts of these salts in ultrapure water, both with and without CO_2_ NBs. For the kinetics study of HBD in sulfate solutions, 300 mL of these prepared solutions was introduced into the high-pressure reactor. Initially, the reactor was purged with CO_2_ to eliminate any residual air. The reactor was then pressurized with CO_2_ until the desired pressure was reached. The operating conditions were set at 274.15 K and 3.58 MPa. To compensate for the pressure decrease caused by CO_2_ consumption, additional CO₂ was periodically introduced by the control valves, restoring the reactor pressure to the set point of 3.58 MPa. The temperature and pressure inside the reactor were continuously monitored for a duration of 400 min. The kinetics of the HBD process were tracked by measuring the CO_2_ gas consumption over time, as described by Eq. 1 [5, 41–43]:

1 $$\Delta {n}_{G}={n}_{G,0}-{n}_{G,t}=\frac{V}{R}\left(\frac{{P}_{0}}{{T}_{0}{z}_{0}}-\frac{{P}_{t}}{{T}_{t}{z}_{t}}\right)$$

where n_G,0_ and n_G,t_ denote the total number of CO_2_ moles in the gas phase at the initial time t = 0 and at any subsequent time t, respectively. It was assumed that the gas volume (V) remained constant throughout the process. Equation 1, *n* = *PV/zRT*, involves parameters such as T (gas temperature), P (pressure), V (volume), and R (universal gas constant), where z represents the compressibility factor calculated through the Peng-Robinson equation of state [44], which allowed us to determine the CO_2_ gas consumption both at the start of the process and at any given time during hydrate formation [43].

For the HBD experiments with the effluent, 500 mL of the effluent, with and without CO_2_ NBs, was introduced into the high-pressure reactor. The same process was repeated to induce hydrate formation in the effluent. Upon completion of the experiment after 180 min, the reactor was depressurized, and the gas hydrates were collected, and vacuum filtered. To minimize hydrate dissociation losses, the hydrates were promptly transferred to a pre-cooled filtration setup after depressurization. Following filtration, the hydrates were dissociated upon exposure to atmospheric conditions under the fume hood, producing water and CO₂ gas, which was then vented through the hood. This is not a significant concern as only a small amount of CO₂ is released at the experimental scale. For industrial applications, the CO₂ gas should be captured, stored, and reused in the process, contributing to a more sustainable and efficient system. The gas hydrate dissociation process can be represented by the following reaction [45]:

$$ {\text{CO}}_{{2}} .{\text{xH}}_{{2}} {\text{O }}\left( {\text{s}} \right) \to {\text{CO}}_{{2}} \left( {\text{g}} \right) + {\text{ xH}}_{{2}} {\text{O}} $$

As the system pressure or temperature moves beyond the hydrate stability conditions (such as atmospheric pressure), the hydrate structure destabilizes, causing the trapped CO_2_ gas to be released while water liquid remains. The resulting water from the melted hydrates and the remaining brine were weighed. This procedure was performed three times to replicate a three-stage operation of the HBD process, aiming for maximum water quality. A conductivity meter (Cond 3110 SET 1, Germany) was used to evaluate desalination efficiency. The desalination efficiency was assessed by comparing the electrical conductivity of the initial solution with that of the desalinated water, since electrical conductivity is representative of the salt concentration, as outlined in Eq. 2 [34, 42, 43, 46–48]:

2 $$Desalination\,efficiency \left(\eta \right) \%=\frac{{C}_{i}-{C}_{f}}{{C}_{i}}\times 100=\frac{{\sigma }_{i}-{\sigma }_{f}}{{\sigma }_{i}}\times 100$$

where *σ*_*i*_ and *σ*_*f*_ represent the electrical conductivity values of the initial solution and the desalinated water, respectively, and both are proportional to the concentration of total dissolved solids, namely C_i_ and C_f_. Hydrate conversion was determined based on the amount of water that participated in the formation of gas hydrates [49], as shown in Eq. 3 [5]:

3 $$Hydrate\,conversion \%=\frac{mass\,of\,water\,converted\,to\,hydrate}{mass\,of\,feed\,solution}\times 100$$

Water recovery was quantified as the ratio of the final desalinated water (m_f_) to the initial feed solution (m_i_), as defined in Eq. 4 [5]:

4 $$Water\,recovery \%=\frac{{m}_{f}}{{m}_{i}}\times 100$$

## Results and Discussion

### Kinetics Study of CO_2_ HBD in Sulfate Solutions

**Fig. 2:**
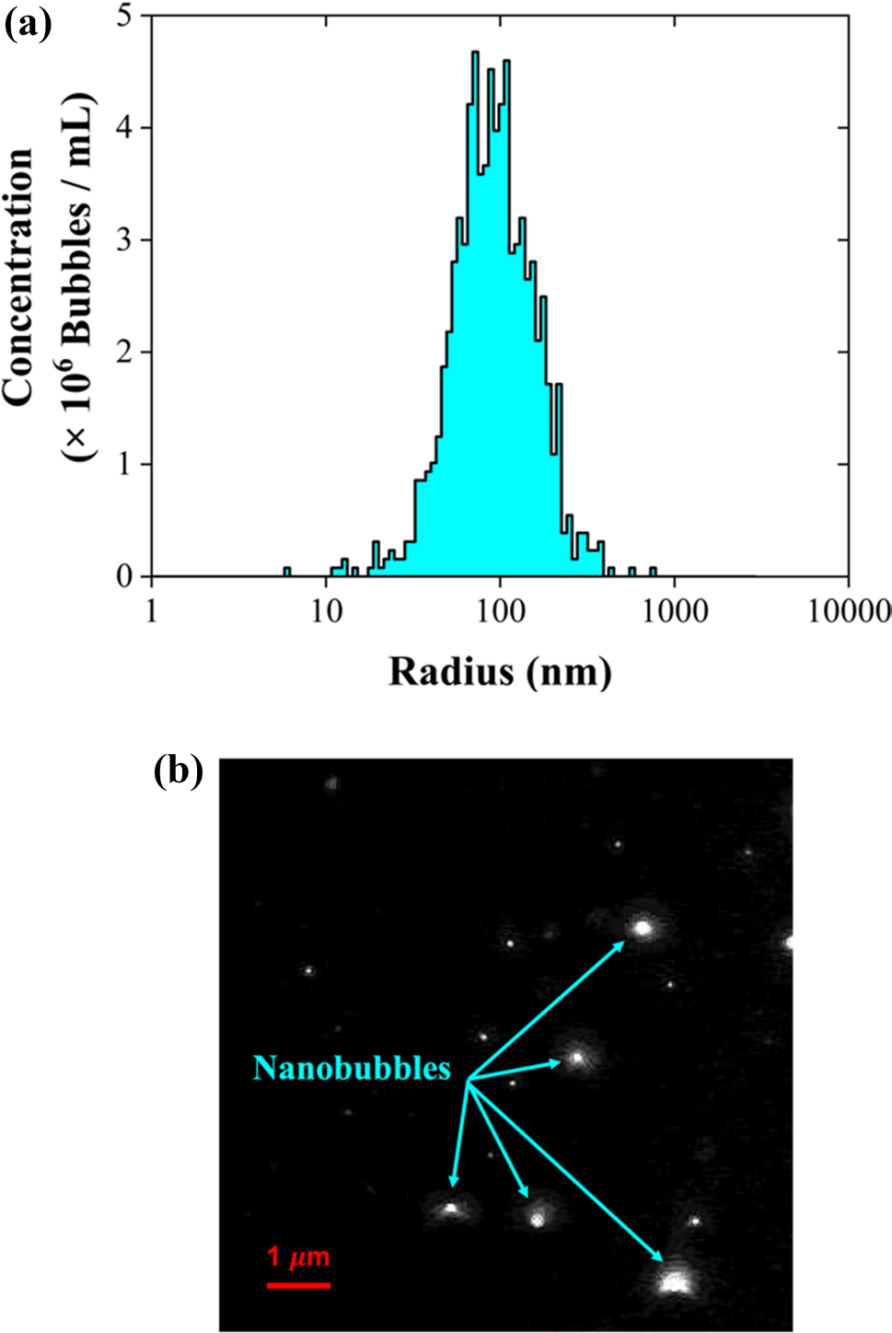
NTA data for CO_2_ NBs: **a** size distribution, and **b** micrograph of a suspension with CO_2_ NBs in ultrapure water

In this study, CO_2_ NBs were used as a novel kinetic promoter in HBD of aqueous sulfate solutions and of a real effluent received from a mining and metals company in Québec, Canada. Figure 2 presents the distribution of CO_2_ NBs sizes and features a micrograph of ultrapure water containing NBs. The micrograph shows CO_2_ NBs as white specks on a black background. The concentration of the NBs was measured at approximately 8.07 ± 0.21 × 10^7^ bubbles/mL, with an average radius of 103.15 ± 2.47 nm. Notably, the blank sample of ultrapure water, prior to the generation of CO_2_ NBs, exhibited no nanosized impurities. According to a previous study [5], these CO₂ NBs have been shown to remain stable for up to 21 days. Furthermore, it was observed that CO₂ NBs maintained their stability even under the conditions of the HBD tests [5].

**Fig. 3:**
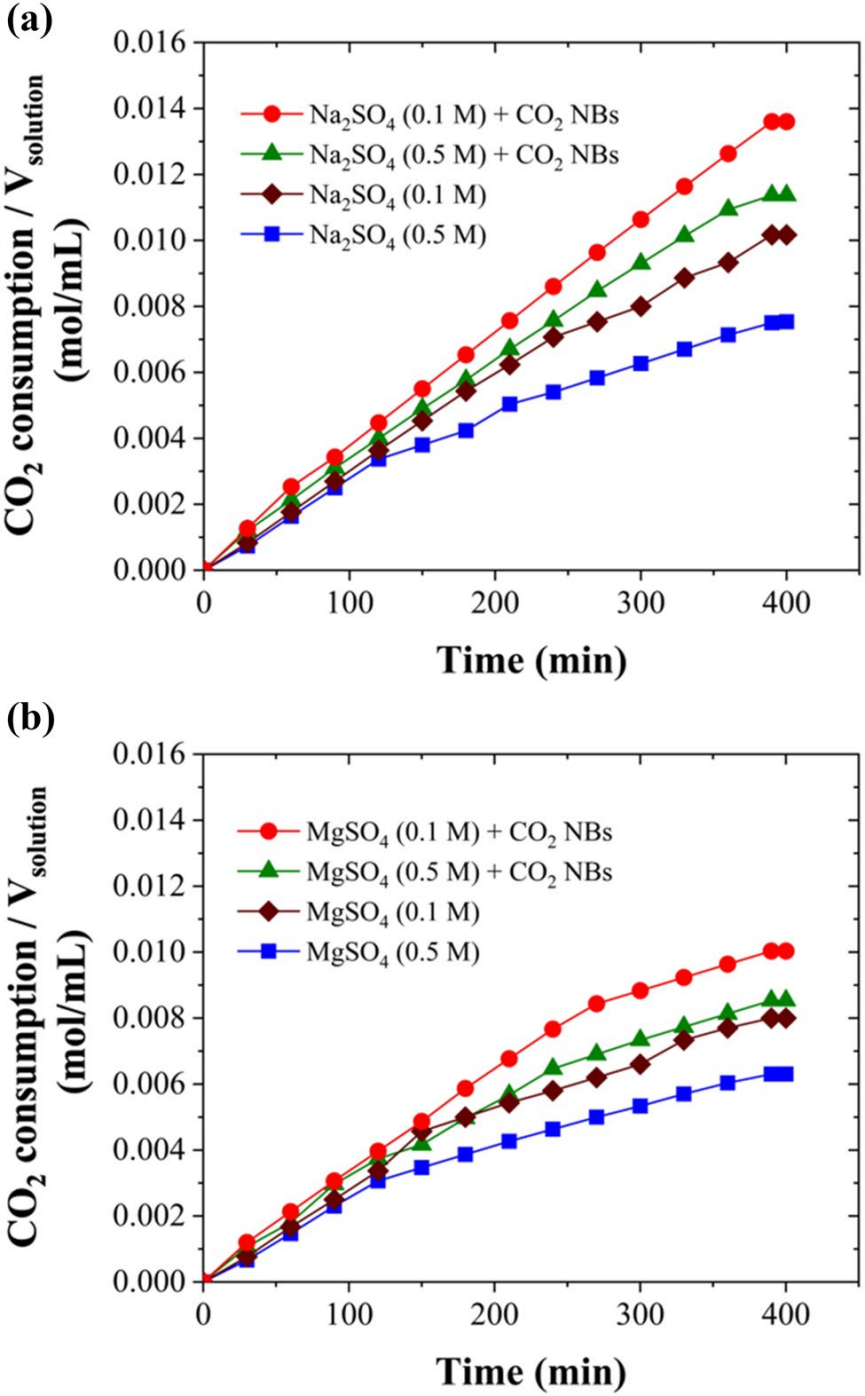
The total CO_2_ gas consumption per volume of solution over time, with and without the presence of CO_2_ NBs in 0.1 and 0.5 M of **a** Na_2_SO4 and **b** MgSO_4_ under stirred conditions. T = 274.15 K, P = 3.58 MPa

Figure 3 shows the CO_2_ gas consumption per unit volume of solution over time, highlighting the process kinetics. The experiment used different concentrations of Na_2_SO_4_ and MgSO_4_, both with and without CO_2_ NBs, under stirred conditions at 300 rpm, a temperature of 274.15 K, and a pressure of 3.58 MPa. Initially, CO_2_ hydrate crystals formed and grew rapidly. After this initial stage, the gas consumption rate decreased. Figures 3a and 3b demonstrate that CO_2_ gas consumption was significantly accelerated in the presence of CO_2_ NBs for both Na_2_SO_4_ and MgSO_4_ solutions. Specifically, CO_2_ gas consumption per unit volume of solution increased from 0.0102 to 0.0136 mol/mL, from 0.0075 to 0.0113 mol/mL, from 0.0080 to 0.0100 mol/mL, and from 0.0063 to 0.0085 mol/mL by the presence of CO_2_ NBs after 400 min for both 0.1 M and 0.5 M of Na_2_SO_4_ and MgSO_4_ solutions, respectively.

The observed promotion mechanism with CO_2_ NBs could be attributed to the memory effect, wherein hydrate crystals tend to form at lower supercooling or supersaturation levels during reformation after dissociation compared to their initial formation [5, 50]. While the precise mechanisms underlying the memory effect remain subject to debate, studies have revealed the presence of NBs in the solution resulting from gas hydrate dissociation [5, 50–53]. Additionally, the gas dissolution hypothesis theorizes that a sufficient concentration of guest molecules in the liquid phase is essential for hydrate formation [36, 50, 54]. However, the hydrophobic nature and low solubility of most gas hydrate former molecules in water present challenges in maintaining a high concentration of guest molecules as hydrate crystals grow, thus impeding the crystallization process [5, 50]. CO_2_ NBs can act as sources of gas hydrate former molecules in the liquid phase, thereby facilitating hydrate crystal formation and growth. Previous research also showed that CO_2_ NBs could boost gas consumption by approximately 65% during CO₂ gas hydrate formation in a 0.5 M NaCl solution after 400 min under the same conditions [5]. This is higher compared to the 51% increase observed in a 0.5 M Na_2_SO_4_ solution reported in this study. According to existing literature, NBs tend to be unstable in highly concentrated saline solutions [40, 55, 56]. The presence of salts in solutions containing NBs can reduce the repulsive electrostatic forces between them, leading to increased coalescence of the bubbles and consequently decreased stability [40]. Additionally, research by Montazeri et al. [40] suggests that even weaker repulsive forces exist between NBs in sulfate solutions compared to chloride solutions, further increasing the likelihood of bubble coalescence. Therefore, sulfate solutions create unfavorable conditions for the stability of NBs. This may lead to a reduced effect of NBs on the process kinetics in sulfate solutions compared to chloride solutions.

Referring to Fig. 3a and b, the kinetics of HBD in Na_2_SO_4_ solutions exhibited higher rates compared to those in MgSO_4_ solutions overall. Salts typically create more challenging conditions for gas hydrate formation and act as inhibitors [6, 57]. This inhibition effect arises from the competition between ions and gas hydrates for water molecules [6]. As ions dissolve, they undergo hydration by water molecules, consequently reducing the available number of hydrogen bonds for hydrate formation [58]. Typically, salts with higher electrical charge and smaller ionic size tend to be stronger inhibitors in hydrate formation [6, 16]. Hence, Mg^2+^ as a divalent ion with a smaller ionic size than Na^+^ may exert a stronger inhibitory effect on the HBD process, potentially resulting in slower kinetics of the process in MgSO_4_ solutions compared to Na_2_SO_4_ solutions.

### Generation of CO_2_ NBs in the Real Effluent

**Fig. 4:**
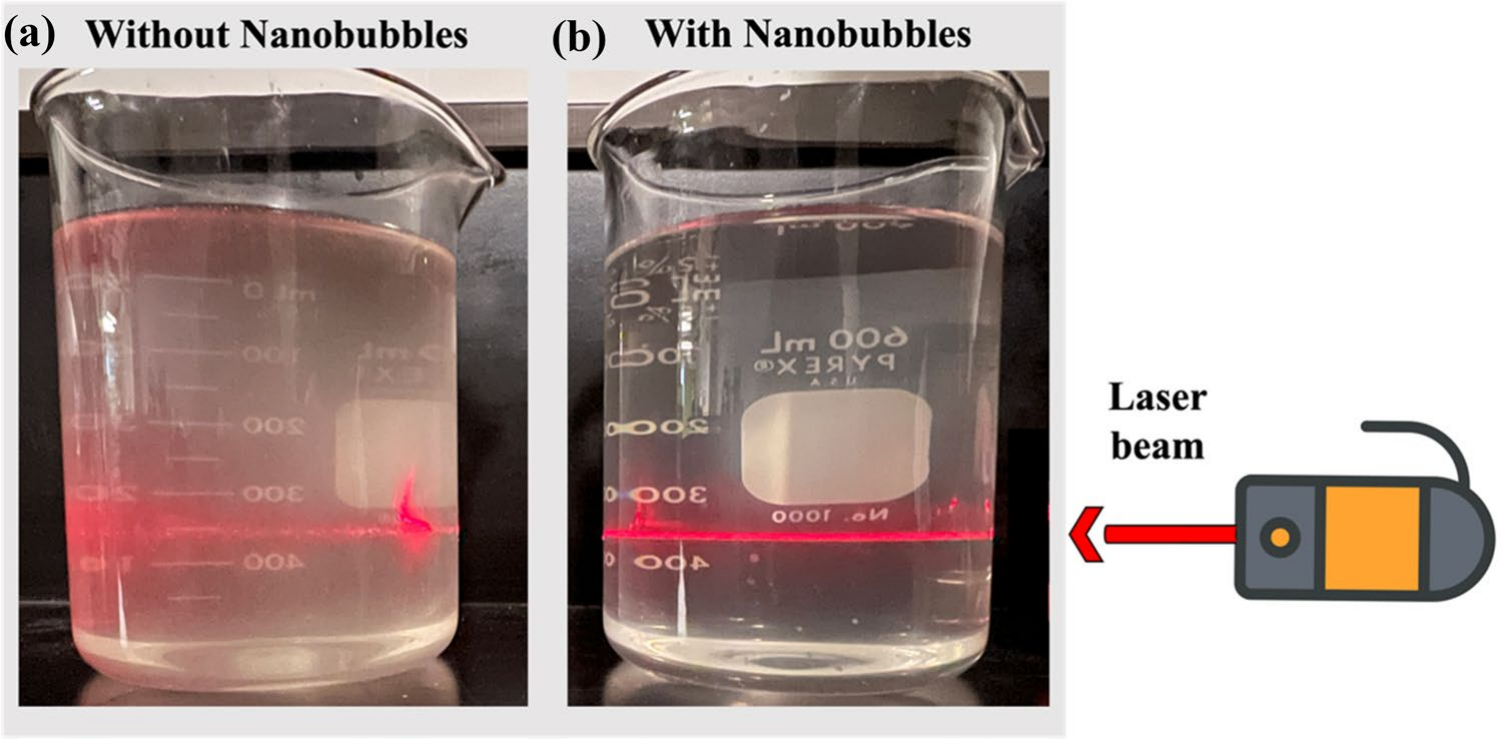
The scattering of a laser beam passing through **a** the effluent and **b** the effluent containing CO_2_ NBs: the Tyndall effect proves the presence of CO_2_ NBs

CO_2_ NBs were introduced as an innovative kinetic promoter in the hydrate-based desalination of a real effluent from the mining and metals industry. To generate CO_2_ NBs in the effluent, the tank was first filled with the effluent, and then the MK1 NanoBubbler™ was operated for 75 min. To verify the presence of NBs in the effluent samples, light scattering technique based on the Tyndall scattering effect was utilized. This well-known phenomenon in colloidal solutions is used to demonstrate the existence of NBs in aqueous solutions [59, 60]. When a laser beam illuminates the solution, the Tyndall effect causes the beam to scatter, making it visible to the naked eye in NBs suspensions, whereas no such scattering can be seen in a clean solution [40, 61]. This observation is supported by numerous studies as evidence of the presence of NBs in aqueous solutions [40, 60, 62, 63]. The application of the Tyndall effect in this study involved illuminating the effluent samples with a red laser beam. As shown in Fig. 4, a bright path in the vertical direction of the incident light can be observed in the effluent samples collected from the MK1 NanoBubbler™ tank, while the light path in the initial sample appears dim and disorganized. This confirms the successful generation and presence of CO_2_ NBs in the effluent.

### Kinetics Study of CO_2_ HBD of the Real Effluent

**Fig. 5:**
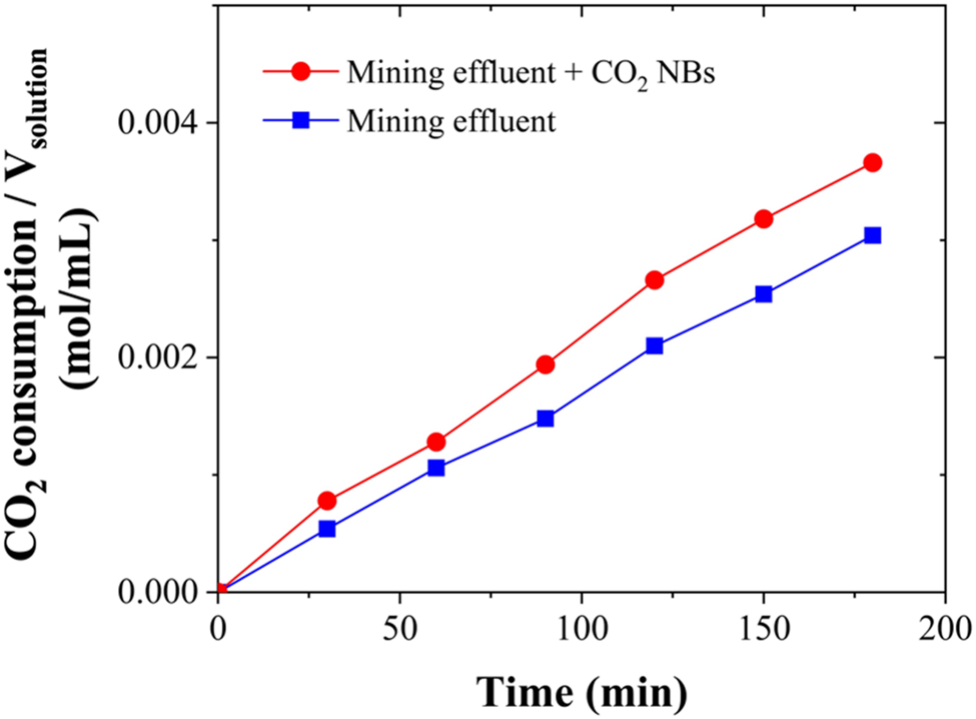
Kinetic study of HBD of the real effluent as CO_2_ gas consumption per unit volume of solution with and without the presence of CO_2_ NBs under stirred conditions (300 rpm), T = 274.15 K, and P = 3.58 MPa

The role of CO_2_ NBs as kinetic promoters of hydrate formation in sulfate solutions was investigated. The findings demonstrated a significant potential of CO_2_ NBs in enhancing the hydrate formation kinetics in aqueous sulfate solutions. Similarly, the influence of CO_2_ NBs on the kinetics of hydrate formation in chloride solutions has been thoroughly examined in a separate study by Montazeri et al. [5]. The effect of CO_2_ NBs on the kinetics of the HBD process of the mining effluent, which contained both chloride and sulfate salts, was also experimentally evaluated in this work, as illustrated in Fig. 5.

Figure 5 shows the rate of CO_2_ gas consumption per unit volume of solution, shedding light on the kinetics of the process. Accordingly, the CO_2_ gas consumption rate was higher in the presence of CO_2_ NBs. The observed enhanced promotion with CO_2_ NBs can be attributed to the memory effect, which was previously described in "Kinetics Study of CO2 HBD in Sulfate Solutions" Section. CO_2_ NBs supply sufficient gas molecules for the hydrate formation process, preventing a significant decrease in dissolved CO_2_ that could otherwise hinder the process. According to Fig. 5, the consumption of CO_2_ gas per unit volume of solution showed an approximate 20% increase, rising from 0.0030 to 0.0036 mol/mL when CO_2_ NBs were present after 180 min, leading to an increase in water recovery from approximately 45.19% to 59.83%.

### Kinetic Model of CO_2_ Hydrate Formation in the Presence of Nanobubbles

At the macroscopic level, the growth rate of gas hydrates in batch reactors is usually determined by calculating the gas consumption rate using pressure and temperature measurements [64]. In semi-continuous systems where the hydrate-forming gases are continuously added, the operating pressure is usually maintained constant and the amount of hydrate forming gas added corresponds to the amount of gas hydrates formed. A comprehensive review of hydrate growth kinetic models has been published a few years ago [64]. Among the existing models, a simple yet elegant model is given by concentration differences and the total surface area of the hydrate particles, as shown in Eq. 5:

5 $$\frac{{dn}_{t}}{dt}=\pi {\mu }_{2}K\left(C-{C}_{eq}\right)/{V}_{L}$$

where n_t_ corresponds to the molar concentration of gas hydrate forming gas (in our case CO_2_), V_L_ is the volume of the liquid phase, μ_2_ is the second moment of particle size distribution and can be readily computed when μ_0_, i.e., the zeroth moment of the particle size distribution and μ_1_,i.e., the first moment of the particle size distribution are also calculated [65], K is the kinetic rate constant, C is the concentration of dissolved CO_2_ in the liquid phase in equilibrium with the partial pressure of CO_2_(g) in the gas phase, which is also the operating pressure if pure CO_2_ is used, and C_eq_ is the concentration of dissolved CO_2_ in the liquid phase in equilibrium at the equilibrium pressure that corresponds to the operating temperature. The initial condition n_t=0_ can be estimated from gas consumption data during the induction period, or it can be considered as an unknown parameter to be estimated together with the kinetic parameter. In this work, since CO_2_ NBs were employed to act as hydrate nucleation points, it follows that we can assume that the μ_0_ is equal to the number of nanobubbles present in solution (known through NTA analysis), thus allowing us to compute μ_2_, which is proportional to the average surface (i.e., radius squared) of the nanobubbles (also known through NTA analysis). Therefore, the kinetic rate can be obtained using Eq. 6:

6 $$\frac{{dn}_{t}}{dt}={K}_{app}{{n}_{t}}^{2/3}$$

where K_app_ is an apparent kinetic rate constant. Integrating this differential equation, we obtain Eq. 7:

7 $${{n}_{t}}^{1/3}={K}_{app}t+{{n}_{t=0} }^{1/3}$$

or Eq. 8:

8 $${n}_{t}={\left[{K}_{app}t+{{n}_{t=0} }^{1/3}\right]}^{3}$$

**Fig. 6:**
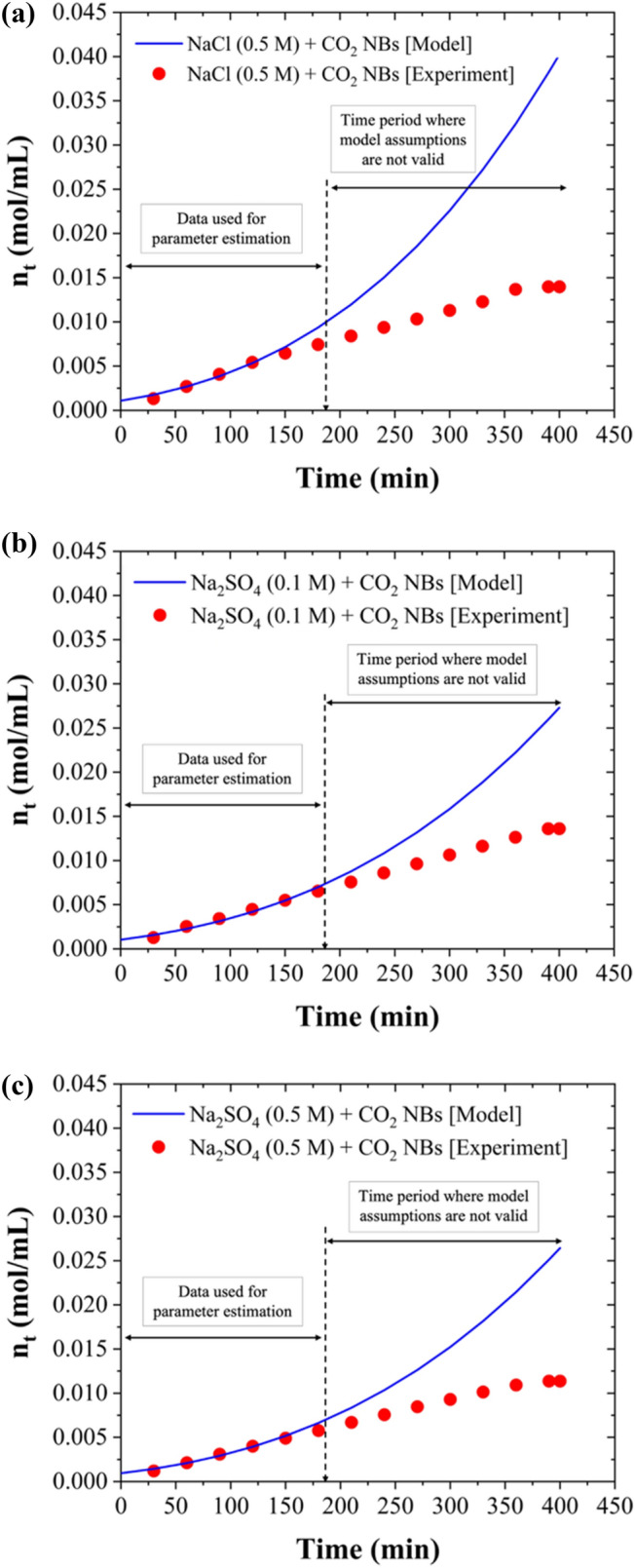
Kinetic model performance for nanobubble-enhanced HBD process of synthesized chloride- and sulfate-: **a** 0.5 M NaCl + CO_2_ NBs (based on data from our previous study [5]), **b** 0.1 M Na_2_SO_4_ + CO_2_ NBs, and **c** 0.5 M Na_2_SO_4_ + CO_2_ NBs (under stirred conditions (300 rpm), T = 274.15 K, and P = 3.58 MPa)

**Fig. 7:**
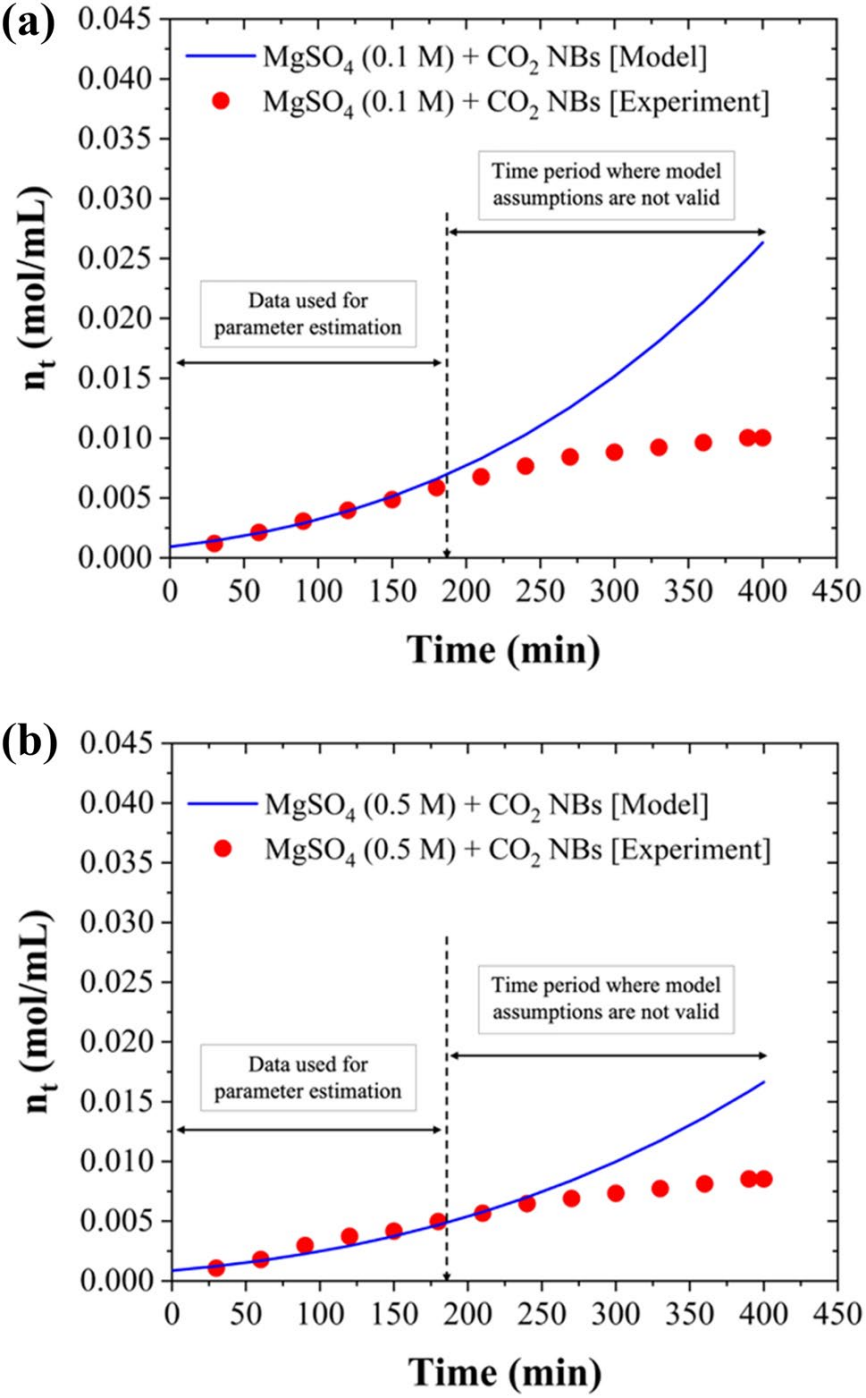
Kinetic model performance for nanobubble-enhanced HBD process of synthesized sulfate-based solutions: **a** 0.1 M MgSO_4_ + CO_2_ NBs and **b** 0.5 M MgSO_4_ + CO_2_ NBs (under stirred conditions (300 rpm), T = 274.15 K, and P = 3.58 MPa)

**Fig. 8:**
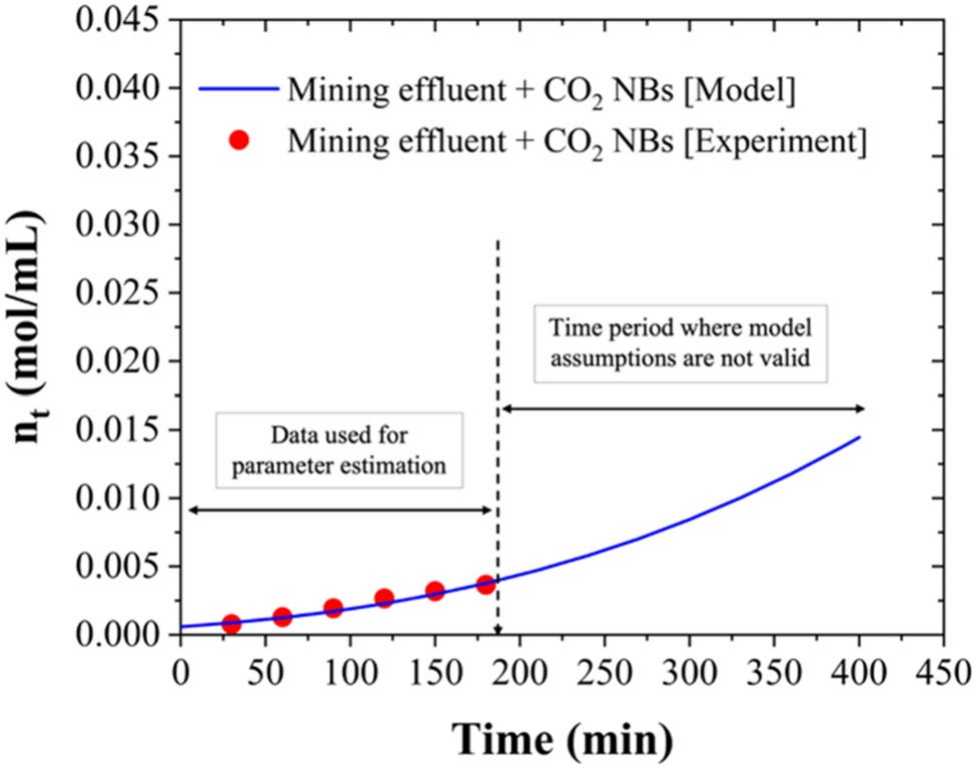
Kinetic model performance for nanobubble-enhanced HBD process of the mining effluent (under stirred conditions (300 rpm), T = 274.15 K, and P = 3.58 MPa)

Figures 6, 7, and 8 present the measured experimental data and the model predictions. The model was developed to match the experimental measurements obtained for the nanobubble-enhanced HBD process of synthesized chloride- and sulfate-based solutions as well as for the real effluent tested in this work. It should be noted that the first 180 min of the experimental gas consumption data were used to estimate the kinetic parameters, as during that time water was in excess compared to the water that had been converted to hydrates. There are several assumptions made in the development of our model, which limit its application to systems with water in excess of the amount converted to hydrates, perfect stirring conditions, and the continuous supply of CO_2_ to be maintained at a constant pressure.

The apparent kinetic rate constants for CO₂ hydrate formation in the synthesized chloride- and sulfate-based solutions as well as in the real effluent were determined through regression analysis at industrially relevant conditions and are presented in Table 2. The results clearly demonstrate that the apparent kinetic rate constant (K_app_) for the hydrate formation is higher in the NaCl solution compared to the Na₂SO₄ solution, highlighting the relatively faster hydrate formation in the chloride system. Generally, chloride ions exhibit a weaker hydration effect compared to sulfate ions [66, 67], meaning more free water molecules are accessible for hydrate formation, which could contribute to the faster formation of CO₂ hydrates. Although both NaCl and Na₂SO₄ are electrolytes, NaCl dissociates into two ions (Na⁺ and Cl⁻), while Na₂SO₄ dissociates into three ions (2Na⁺ and SO₄^2^⁻). The resulting higher ionic strength in Na₂SO₄ solutions [40] can lead to increased solution viscosity, potentially slowing down the diffusion of CO₂ molecules to the hydrate formation sites and thus inhibiting the formation rate. Furthermore, the presence of salts reduces water activity, contributing to their inhibitory effects on hydrate formation [6]. In sulfate solutions, the lower water activity compared to chloride solutions leads to more restricted movement of both water molecules and CO₂ gas. This limitation hinders the interaction of CO₂ with water, thereby slowing down the nucleation and growth of hydrates.

**Table 2:** Summary of the kinetic parameters for CO₂ hydrate formation in synthesized solutions and the real effluent

Solution	K_app_ (1/min)(mol/mL)^1/3^	n_t=0_ (mol/mL)	R^2^ (*)
0.5 M NaCl + CO_2_ NBs	0.000555	0.001083	0.95
0.1 M Na_2_SO_4_ + CO_2_ NBs	0.000503	0.001033	0.97
0.5 M Na_2_SO_4_ + CO_2_ NBs	0.000478	0.000939	0.97
0.1 M MgSO_4_ + CO_2_ NBs	0.000482	0.000927	0.98
0.5 M MgSO_4_ + CO_2_ NBs	0.000449	0.000865	0.95
Mining effluent + CO_2_ NBs	0.000419	0.000584	0.97

(*) The R^2^ in the table have been computed in the time period [0, 180 min]

Additionally, the kinetic rate constants are higher in Na_2_SO_4_ solutions compared to MgSO_4_ solutions, aligning with the findings in Fig. 3, showing a stronger inhibition effect of Mg^2^⁺ compared to Na⁺ on the HBD process. Moreover, the apparent kinetic rate constant for the HBD of the mining effluent was lower than the K_app_ values calculated for the synthesized solutions, indicating that the kinetics of the process are slower in the mining effluent. This slower rate is likely due to the complex composition of the mining effluent, which contains multiple salts, including chlorides and sulfates, that collectively contribute to a greater inhibition effect and reduced kinetics of the HBD process.

Finally, it should be noted that K_app_ that we estimated for our experiments incorporates besides the growth kinetic rate constant, several other process parameters, namely, the number of nucleation sites (μ_0_, which is equal to the number of NBs present in the solution), the volume of the liquid phase, and the driving force (C-C_eq_), which was assumed to be maintained constant by the operator.

### Key HBD Parameters for the Real Effluent with and Without CO_2_ NBs

**Fig. 9:**
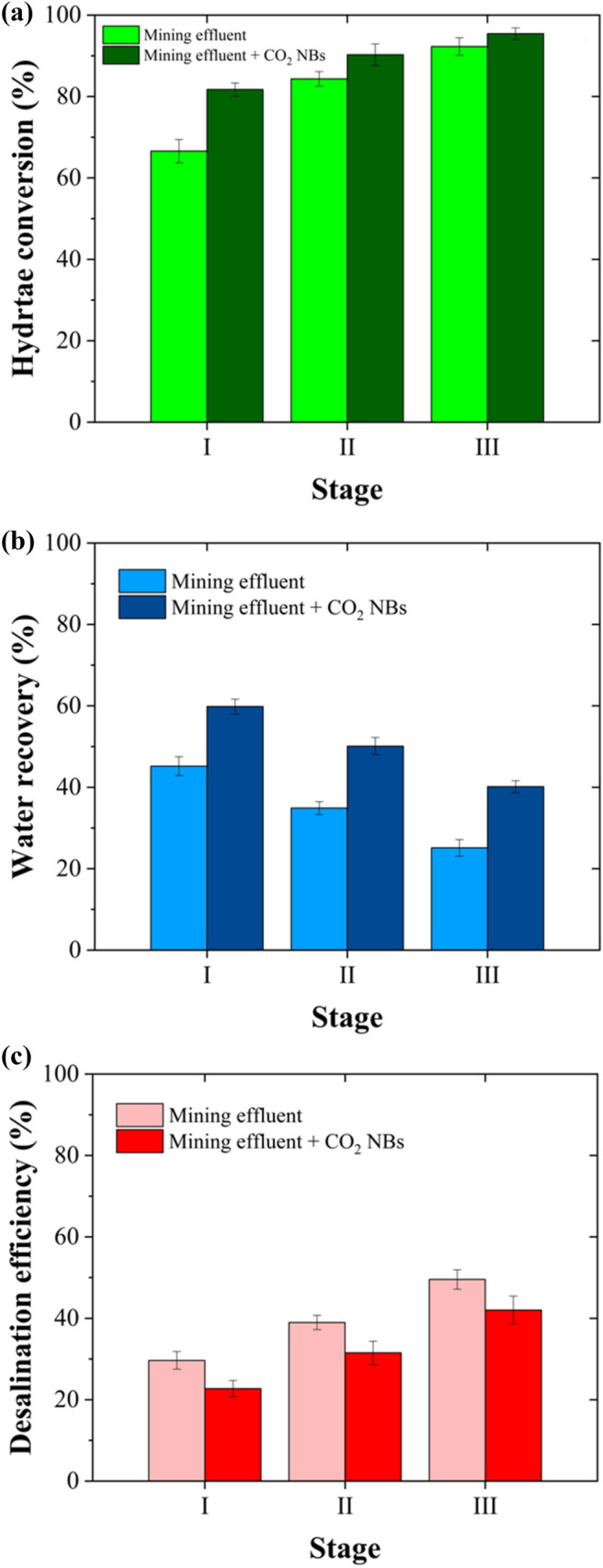
Critical aspects of the HBD process for real mining effluent, both with and without CO_2_ NBs as kinetic promoters, include **a** hydrate conversion, **b** water recovery, and **c** desalination efficiency. Over a 3-hour multistage HBD process, hydrate conversion and desalination efficiency gradually increased, though water recovery decreased. The presence of CO_2_ NBs resulted in higher water recovery compared to the process without them

The three stages of HBD for the real effluent, utilizing CO_2_ gas as the gas hydrate former and CO_2_ NBs as the kinetic promoter, were conducted over a period of 180 min per stage. The key HBD parameters, specifically hydrate conversion, water recovery, and desalination efficiency, were measured and are presented in Fig. 9. Hydrate conversion rose from 66.59 ± 2.86% to 92.28 ± 2.17% without CO_2_ NBs and from 81.72 ± 1.62% to 95.44 ± 1.43% with CO_2_ NBs, as the number of stages increased from one to three (Fig. 9a). This enhancement can be attributed to the increased driving force for hydrate formation, which results from the reduction in the salinity of the solutions at the end of each stage [49]. Figure 9b shows the water recovery for all stages. After three stages, the final water recovery increased from 25.13 ± 2.04% to 40.16 ± 1.43% in the presence of CO_2_ NBs after 180 min. The water recovery value achieved in this study after three stages, with CO_2_ NBs as the kinetic promoter (40.16 ± 1.43%), is noteworthy and comparable to the performance of RO, which is widely used in desalination [6].

It is important to note that the quality of the produced water should also be considered when evaluating the performance of a desalination process. Therefore, the desalination efficiency of the HBD was calculated for the solutions tested, both with and without the presence of CO_2_ NBs, at each stage. As shown in Fig. 9c, salts were steadily removed from the recovered water at each stage of the HBD process. The final desalination efficiency in the presence of CO_2_ NBs was 42.03 ± 3.43% compared to 49.54 ± 2.39% obtained in the effluent without the use of CO_2_ NBs. The presence of CO_2_ NBs accelerates the formation of hydrate crystals, which in turn enhances the probability of salt entrapment among these crystals, unlike solutions without CO_2_ NBs [5]. Consequently, this phenomenon could lead to a slight decrease in desalination efficiency; however, this is coupled with a significant boost in water recovery (from 25.13 ± 2.04% to 40.16 ± 1.43%). The findings indicate that achieving clean water recovery from complex solutions like real mining and metal processing effluents is feasible through HBD technology. Moreover, the kinetics of the process can be considerably enhanced with the presence of NBs, rendering it appealing for industrial applications.

## Conclusions

This study investigated CO_2_ HBD technology for clean water recovery from synthetic sulfate solutions as well as from a real effluent from the mining and metals industry, which contained both chloride and sulfate salts. The kinetic behavior of the HBD process was investigated for the first time in various concentrations of Na_2_SO_4_ and MgSO_4_ solutions, both with and without CO_2_ NBs as kinetic promoter. The results demonstrated that CO_2_ NBs can notably enhance the kinetics of the process in sulfate solutions by increasing the CO_2_ consumption per unit volume of solution. A kinetic model of CO_2_ hydrate formation in the presence of nanobubbles was developed. Following this, the CO_2_ NB-boosted HBD process was evaluated as a novel and energy-efficient method for water recovery from an actual effluent from the mining and metals industry. Critical parameters of the HBD process, including hydrate conversion, water recovery, and desalination efficiency, were documented. The kinetics of the process were examined over a hydrate formation period of 180 min. Hydrate conversion across the three stages was observed to increase from 81.72 ± 1.62% to 95.44 ± 1.43% in the samples containing CO_2_ NBs. Accordingly, CO_2_ consumption per unit volume of solution was observed to rise by roughly 20% with the introduction of CO_2_ NBs. Moreover, water recovery increased from 25.13 ± 2.04% to 40.16 ± 1.43% in the presence of CO_2_ NBs, providing further evidence of their promoting effect. The water recovery yield obtained is comparable to what is typically encountered in conventional desalination processes. Moreover, both with and without CO_2_ NBs, desalination efficiencies of 42.03 ± 3.43% and 49.54 ± 2.39%, respectively, were attained, suggesting that the kinetic promotion does not significantly impact the desalination efficiency of the process. The application of the CO_2_ HBD method, coupled with the promotion provided by CO_2_ NBs, presents valuable findings for the further advancement of HBD technology, particularly for the recovery of clean water for reuse in the mining, mineral, and metal processing industry.
